# Retinal OCTA parameters and the risk of worsening kidney function in patients with type 2 diabetes: a longitudinal cohort study

**DOI:** 10.3389/fendo.2026.1924491

**Published:** 2026-09-14

**Authors:** Ying Huang, Gong Chen, Qianhua Fang, Zhiyun Zhao, Juan Shi, Ying Peng, Xuetong Wang, Cong Liu, Xing Wei, Lin Sun, Zhuomeng Hu, Yuening Xu, Ping Xue, Jie Hong, Weiqiong Gu, Na Li, Yifei Zhang, Xi Shen, Weiqing Wang

**Affiliations:** 1 Department of Ophthalmology, Ruijin Hospital, Shanghai Jiao Tong University School of Medicine, Shanghai, China; 2 Department of Endocrine and Metabolic Diseases, Shanghai Institute of Endocrine and Metabolic Diseases, Ruijin Hospital, Shanghai Jiao Tong University School of Medicine, Shanghai, China; 3 National Clinical Research Center for Endocrine and Metabolic Diseases, Key Laboratory for Endocrine and Metabolic Diseases of the National Health Commission of the PR China, Ruijin Hospital, Shanghai Jiao Tong University School of Medicine, Shanghai, China

**Keywords:** optical coherence tomography angiography, renal function decline, retinal microvasculature, type 2 diabetes, vessel density

## Abstract

**Aims:**

To determine whether retinal optical coherence tomography angiography (OCTA) parameters are associated with subsequent worsening kidney function (WKF) in patients with type 2 diabetes (T2D) and to assess their relationship with early renal function trajectory.

**Methods:**

In this prospective cohort study, 180 patients with T2D underwent macular OCTA imaging and were followed for renal outcomes (median follow-up, 37 months). Cross-sectional associations between OCTA parameters and systemic biomarkers were examined. Multivariable Cox models adjusted for age, sex, diabetes duration, HbA1c, HDL-c, and baseline estimated glomerular filtration rate (eGFR) evaluated associations with WKF. eGFR slope over the first 12 months was estimated using linear mixed-effects models in 141 participants. Sensitivity analyses further evaluated image quality distributions, ocular comorbidities, single-eye selection, and additional adjustments for baseline renal/hemodynamic parameters. Exploratory mediation analysis assessed whether early eGFR slope accounted for OCTA-outcome associations.

**Results:**

Forty-two participants (23%) developed WKF. Several OCTA parameters correlated with systemic and renal biomarkers in unadjusted analyses but were not significant after multivariable adjustment. In primary adjusted Cox models, nasal inner-ring superficial vessel density (NI-SVD) was independently associated with WKF (HR, 1.10 per 1% increase; 95% CI, 1.02-1.19; p=0.013). Early eGFR slope (-1.53 ± 1.38 mL/min/1.73 m^2^/year) was strongly associated with WKF (HR, 0.70; 95% CI, 0.54-0.89; p=0.004). NI-SVD remained significant after adjustment for early eGFR slope (HR, 1.11; 95% CI, 1.02-1.21; p=0.021). Sensitivity analyses indicated that the association between NI-SVD and WKF remained consistent with the primary analysis. Mediation analysis did not demonstrate a statistically significant indirect effect (indirect HR = 1.02, 95% CI: 0.996-1.050).

**Conclusions:**

NI-SVD, a parafoveal OCTA metric was independently associated with WKF in T2D. This association was not explained by early renal functional decline, suggesting that retinal microvascular alterations are independently linked to long-term renal prognosis in T2D.

## Background

The global burden of diabetes mellitus continues to increase, with type 2 diabetes (T2D) accounting for more than 90% of cases worldwide (1). Diabetic retinopathy (DR) and diabetic kidney disease (DKD) are major chronic microvascular complications of T2D. DKD affects nearly 40% of individuals with diabetes and is a leading cause of end-stage renal disease, as well as a major contributor to cardiovascular morbidity and mortality (2, 3). Early identification of individuals at risk for progressive renal function decline therefore remains a critical clinical objective.

Current assessment of DKD primarily relies on serum creatinine-based estimated glomerular filtration rate (eGFR) and urinary albumin excretion, while kidney biopsy serves as the histopathological reference standard in selected cases. Although eGFR is widely used for monitoring renal function and risk stratification, it reflects established functional impairment and may not fully capture early microvascular injury (4, 5). These limitations underscore the need for complementary, noninvasive markers that can detect early vascular alterations before overt renal dysfunction becomes apparent.

The retina and kidney share similar microvascular architecture, developmental origins, and regulatory pathways, supporting a close pathophysiological link between ocular and renal microvascular damage (6–9). At the cellular and structural level, both organs rely on dense microvascular networks maintained by crucial cellular interactions-specifically, endothelial cell-pericyte interactions in the inner blood-retinal barrier (iBRB) and endothelial cell-podocyte interactions in the glomerular filtration barrier (10). Due to their high metabolic demand and limited/specialized vascular supply, these terminal microvascular beds are exceptionally sensitive to metabolic dysregulation, hyperglycemia-induced oxidative stress, and tissue hypoxia (11). Under chronic glycemic and inflammatory stress, retinal pericytes and renal podocytes display parallel vulnerability; loss or injury of these perivascular supportive cells leads to the breakdown of both the iBRB and the glomerular filtration barrier, increased vascular permeability, capillary dropout, and capillary rarefaction in both tissues (10, 11). The optical transparency of ocular tissues thus provides a unique, noninvasive window for directly visualizing these shared microvascular alterations *in vivo*.

Recently, deep-learning models and foundation frameworks applied to color fundus photographs and retinal images have demonstrated remarkable capabilities for identifying chronic kidney disease and diabetic kidney disease (DKD), as well as assisting longitudinal clinical assessment (12–14). While these end-to-end artificial intelligence approaches (AI) offer holistic screening capabilities, they often function as “black boxes” lacking biological interpretability. Optical coherence tomography angiography (OCTA) can complement these AI approaches by delivering layer-segmented, mechanistically transparent insights into the underlying microvasculature. Given that retinal capillary perfusion naturally exhibits pronounced spatial and temporal heterogeneity to match localized metabolic demands, quantifying these subtle perfusion dynamics provides direct characterization of early microvascular impairment (15). Investigating granular OCTA parameters thus provides a biologically transparent, layer-specific perspective that enriches black-box AI systems by deepening our pathophysiological understanding of the eye-kidney axis. However, to date, most studies examining the relationship between OCTA parameters and renal involvement in T2D have been cross-sectional and focused on baseline renal function measures (16–18). Longitudinal evidence linking OCTA-derived parameters to subsequent renal outcomes remains limited. Accordingly, the present study aimed to examine cross-sectional associations between representative macular OCTA parameters and systemic biomarkers, and evaluate the associations between baseline OCTA parameters and subsequent worsening kidney function (WKF) in patients with T2D. In addition, we explored the potential role of early eGFR trajectory in these associations.

## Methods

### Study design and participants

This single-centre prospective observational cohort study enrolled adults with type 2 diabetes mellitus (T2D) from the National Metabolic Management Center (MMC) at Ruijin Hospital, Shanghai, China (19). The details of the MMC initiative have been published in prior literature (20, 21). Participants who underwent baseline OCTA imaging between March 2021 and November 2021 were consecutively included.

All participants were aged ≥18 years and had not received prior ocular treatment. Written informed consent was obtained from all individuals. The study was approved by the Institutional Review Board of Ruijin Hospital (Approval No. KY2020-183) and conducted in accordance with the Declaration of Helsinki. Staging of retinopathy was based on color fundus photographs analyzed by trained ophthalmologists according to the International Clinical Diabetic Retinopathy Disease Severity Scale (ICDR scale) (22). Exclusion criteria were as follows: best-corrected visual acuity worse than Snellen 20/40 and/or intraocular pressure ≥ 21mmHg; high refractive error, such as axial length (AL) ≥ 26.0 mm, spherical degree ≤ -6 diopters (D), and astigmatism ≥ 3D; any ocular disorder other than DR (e.g., glaucoma, amblyopia, severe cataract, eye trauma, age-related macular degeneration, retinal vascular occlusion, macular epi-membrane and endophthalmitis); any history of intraocular procedures or refractive surgery; any history of renal disease other than DKD. For bilateral eligible eyes, the eye with more severe DR was selected to reflect greater microvascular involvement; if DR severity was equal, the right eye was chosen.

### Ophthalmic examination and OCTA acquisition

OCTA parameters were acquired using the RTVue-XR Avanti spectral-domain optical coherence tomography system equipped with AngioVue software (Version 2018.1.1.63; Optovue, Fremont, CA, USA) in High-Definition (HD) 6.0 x 6.0 mm retina scan mode following pupil dilation. Projection artifacts were automatically removed using the software’s integrated 3D Projection Artifact Removal algorithm (23, 24). Automated retinal layer segmentations were independently inspected across all scans by trained ophthalmologists masked to outcome status to verify correct boundary delineation. Vessel density (VD) was defined as the proportion of perfused vasculature area within the region of interest. The retinal microvasculature was segmented into two layers: the superficial vascular complex (SVC), from the internal limiting membrane (ILM) to 10 μm below the inner plexiform layer (IPL). and the deep vascular complex (DVC): from 10 μm below the inner plexiform layer to 10 μm above the outer plexiform layer (OPL). Retinal thickness was segmented into two layers: the inner retina, from the ILM to the IPL; and the outer retina, from the IPL to the retinal pigment epithelium (RPE). Quantitative parameters were extracted according to the Early Treatment Diabetic Retinopathy Study (ETDRS) grid (25), which was divided into three concentric regions: the center (1 mm diameter), the inner ring (1–3 mm), and the outer ring(3–6 mm). Images with motion artefacts, segmentation errors, signal strength index <40, or quality index <6 were excluded.

### Clinical and laboratory assessment

Standardized questionnaire assessments and comprehensive clinical and laboratory evaluations were conducted for all participants at both enrollment and follow-up visits, with data systematically recorded in the MMC-designated electronic medical record system. The methodologies of the MMC project have been comprehensively delineated in ClinicalTrials.gov (NCT03811470). Mean arterial pressure (MAP) was determined using the formula: diastolic blood pressure plus one-third of the pulse pressure. eGFR was calculated using the 2009 Chronic Kidney Disease Epidemiology Collaboration (CKD-EPI) creatinine equation (26). Serum creatinine was measured by an enzymatic assay on the Beckman Coulter AU5800 automatic clinical chemistry analyzer. Other laboratory tests of blood samples included lipid profiles, liver function, blood routine test.

### Definition of outcome

The primary outcome was worsening kidney function (WKF), defined as a ≥20% decline from baseline eGFR during follow-up. Time-to-event was calculated from baseline OCTA examination to first occurrence of WKF or last follow-up.

### Statistical analysis

Missing values were imputed using random forest methods prior to analysis, implemented via the missForest package (version 1.5) in R (version 4.1.2). Missing data on our study variables ranged from 0% to 1%. We used the completed data for the following analysis. For categorical variables, descriptive statistics were presented as frequencies and proportions, while continuous variables were reported as median (Q1-Q3) or mean (SD). Normality was tested using the Shapiro-Wilk test. Median follow-up duration was calculated using the reverse Kaplan-Meier.

Cross-sectional associations between OCTA parameters and systemic variables were evaluated using Pearson or Spearman correlation analyses. Multivariable linear regression models adjusting for age, sex, and diabetes duration were constructed to assess independent associations.

Given the high dimensionality of OCTA features, least absolute shrinkage and selection operator (LASSO) regression was applied to identify candidate markers associated with WKF. In 10-fold cross-validation, the strict 1-standard-error rule (lambda_1se) yielded an empty model while lambda_min selected 19 features; thus, a parsimonious regularization parameter (lambda = 0.0593) within lambda_min, lambda_1se was selected to balance model complexity and performance, retaining 3 robust candidate features. Feature selection stability was evaluated via 1000 bootstrap iterations under this specified lambda, identifying the top selected markers. Selected variables were entered into multivariable Cox models adjusting for established clinical risk factors. Hazard ratios (HRs) and 95% confidence intervals (CIs) were reported. The estimated early eGFR slope was subsequently incorporated into Cox models to evaluate whether OCTA associations were independent of early renal functional decline. To ensure temporal separation between early eGFR slope estimation and subsequent WKF ascertainment, a 12-month landmark analysis was performed. The analysis was restricted to participants who had at least two eGFR measurements and remained free of WKF during the first 12 months, with a total follow-up duration exceeding 12 months (n=141). Follow-up time was recalculated from the 12-month landmark to the first subsequent occurrence of WKF or the end of follow-up. An exploratory mediation analysis using a bootstrap approach (1,000 resamples) was performed to assess whether early eGFR slope contributed to the association between OCTA parameters and WKF. Statistical analyses were performed in R (The R Foundation, Vienna, Austria, version 4.1.2). A two-sided P < 0.05 was considered statistically significant.

### Sensitivity analysis

To address the potential influence of baseline kidney function, albuminuria, and blood pressure while limiting model complexity given the 42 WKF events, we performed a parsimonious sensitivity analysis in the full cohort (n=180) and slope-analysis sub-cohort (n=141). Sensitivity models included diabetes duration, HbA1c, HDL-c, NI-SVD, baseline eGFR, MAP, and natural log-transformed UACR [ln(UACR)]. Image quality metrics (Signal Strength Index [SSI] and Quality Index [QI]) were compared between participants with and without WKF events. Second, multivariable Cox models for NI-SVD were evaluated under four sensitivity conditions: (1) excluding participants with diabetic macular edema (DME), (2) additional adjustment for baseline DR severity grade, (3) additional adjustment for baseline AL, and (4) restricting the analysis to a prespecified single eye (the right eye of each participant). To evaluate potential non-linear associations, restricted cubic splines (3 knots) were fitted, with hazard ratios additionally calculated per 1-SD increase in NI-SVD while adjusting for image quality and DR severity.

## Results

### Study population and baseline characteristics

Between March and November 2021, 208 patients were prospectively screened, of whom 190 (91%) met the eligibility criteria, and ten (5%) were lost to follow-up. Ultimately, 180 patients were included in the study. A flowchart illustrating the patient selection process is presented in **Figure 1**.

**Figure 1:**
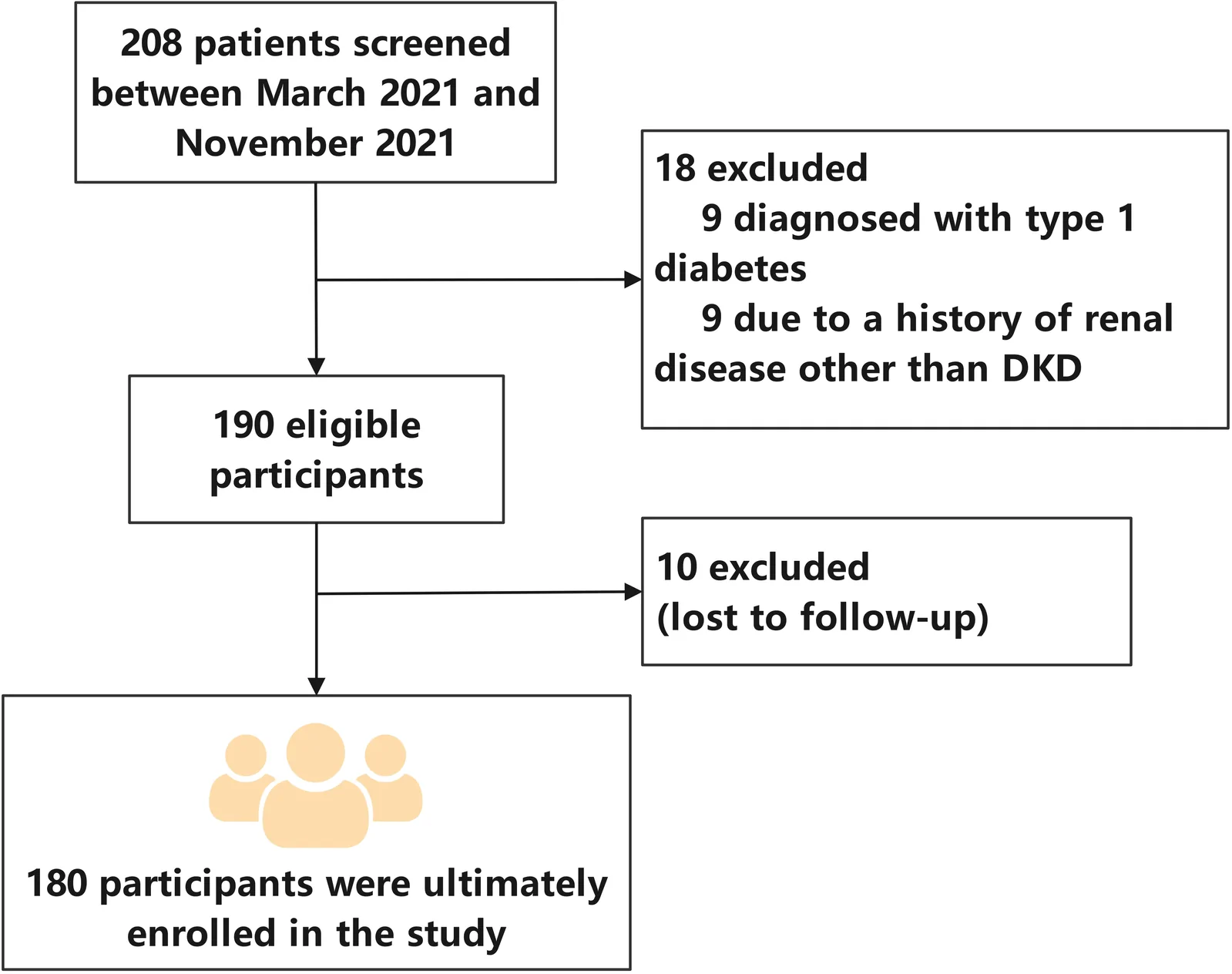
Patient selection process.

The demographic and clinical characteristics at baseline are shown in **Table 1**. The median duration of follow-up was 37 months (95% CI: 36–38 months). Forty-two (23%) of the 180 patients experienced WKF.

**Table 1:** Baseline characteristics.

Characteristics	Level	Overall	No event	WKF event	*P*
n		180	138	42	
Demographics					
Age, years [median (IQR)]		60.00 [50.00, 65.00]	59.50 [50.25, 65.00]	61.00 [49.25, 65.75]	0.802
Sex (%)	Female	82 (45.56)	69 (50.00)	13 (30.95)	0.046
	Male	98 (54.44)	69 (50.00)	29 (69.05)	
Diabetes Duration, months [median (IQR)]		126.77 [68.72, 178.82]	121.30 [56.70, 174.35]	141.10 [81.61, 194.38]	0.088
Hemodynamics & metabolic factors					
MAP, mmHg [mean (SD)]		89.95 (11.18)	89.05 (11.04)	92.90 (11.25)	0.051
UACR, mg/mmol		2.50 [1.17, 4.78]	2.09 [1.07, 3.14]	3.57 [2.17, 15.62]	<0.001
eGFR, mL/min/1.73 m²		94.13 [86.12, 104.55]	95.38 [88.04, 105.72]	89.54 [85.42, 100.13]	0.033
HbA1c, % [median (IQR)]		7.30 [6.70, 8.30]	7.15 [6.50, 8.00]	7.75 [7.20, 8.88]	0.003
HDL-c, mmol/L [median (IQR)]		1.17 [0.99, 1.41]	1.23 [1.03, 1.50]	1.05 [0.87, 1.19]	0.001
Key OCTA parameters					
NI-SVD, % [median (IQR)]		51.51 [48.05, 54.02]	51.39 [47.33, 53.84]	52.12 [49.66, 54.18]	0.086
C-IV, mm^3^ [median (IQR)]		0.039 [0.035, 0.044]	0.040 [0.036, 0.044]	0.036 [0.033, 0.043]	0.025
OI-RT, μm [median (IQR)]		271.35 [259.55, 280.72]	270.70 [258.53, 280.03]	274.35 [268.72, 286.02]	0.035
Staging of Retinopathy (%)					
Normal		71 (39.44)	55 (39.86)	16 (38.10)	0.144
Mild NPDR		58 (32.22)	49 (35.51)	9 (21.43)	
Moderate NPDR		42 (23.33)	29 (21.01)	13 (30.95)	
Severe NPDR		8 (4.44)	4 (2.90)	4 (9.52)	
PDR		1 (0.56)	1 (0.72)	0 (0.00)	

WKF, worsening kidney function; MAP, mean arterial pressure; UACR, urinary albumin-to-creatinine ratio; eGFR, estimated glomerular filtration rate; HbA1c, glycated hemoglobin; HDL-c, high-density lipoprotein cholesterol; NI-SVD, nasal inner superficial vessel density; C-IV, central inner macular volume (ILM-IPL, central 1-mm zone); OI-RT, inferior outer-ring retinal thickness (ILM-RPE); NPDR, nonproliferative diabetic retinopathy; PDR, proliferative diabetic retinopathy.

### Cross-sectional associations between OCTA parameters and systemic parameters

To investigate the systemic relevance of retinal structural and perfusion characteristics, we examined the cross-sectional correlations between representative macular OCTA parameters and systemic biomarkers at baseline in patients with T2D. Vessel density in both the SVC (SVD) and DVC (DVD)was assessed as a key perfusion parameter. After Benjamini-Hochberg correction for multiple comparisons, several significant associations were identified between retinal OCTA parameters and systemic clinical or metabolic variables in unadjusted analyses.

As shown in **Figure 2A**, both SVD and DVD demonstrated consistent negative correlations with age and diabetes duration. In particular, SVD in perifovea and DVD in perifovea were significantly inversely correlated with age (r = -0.32 and -0.30, respectively; both p < 0.01) (**Figure 2B**), while DVD in whole ETDRS exhibited a negative association with diabetes duration (r = -0.30, P = 0.006) (**Figure 2C**).

**Figure 2:**
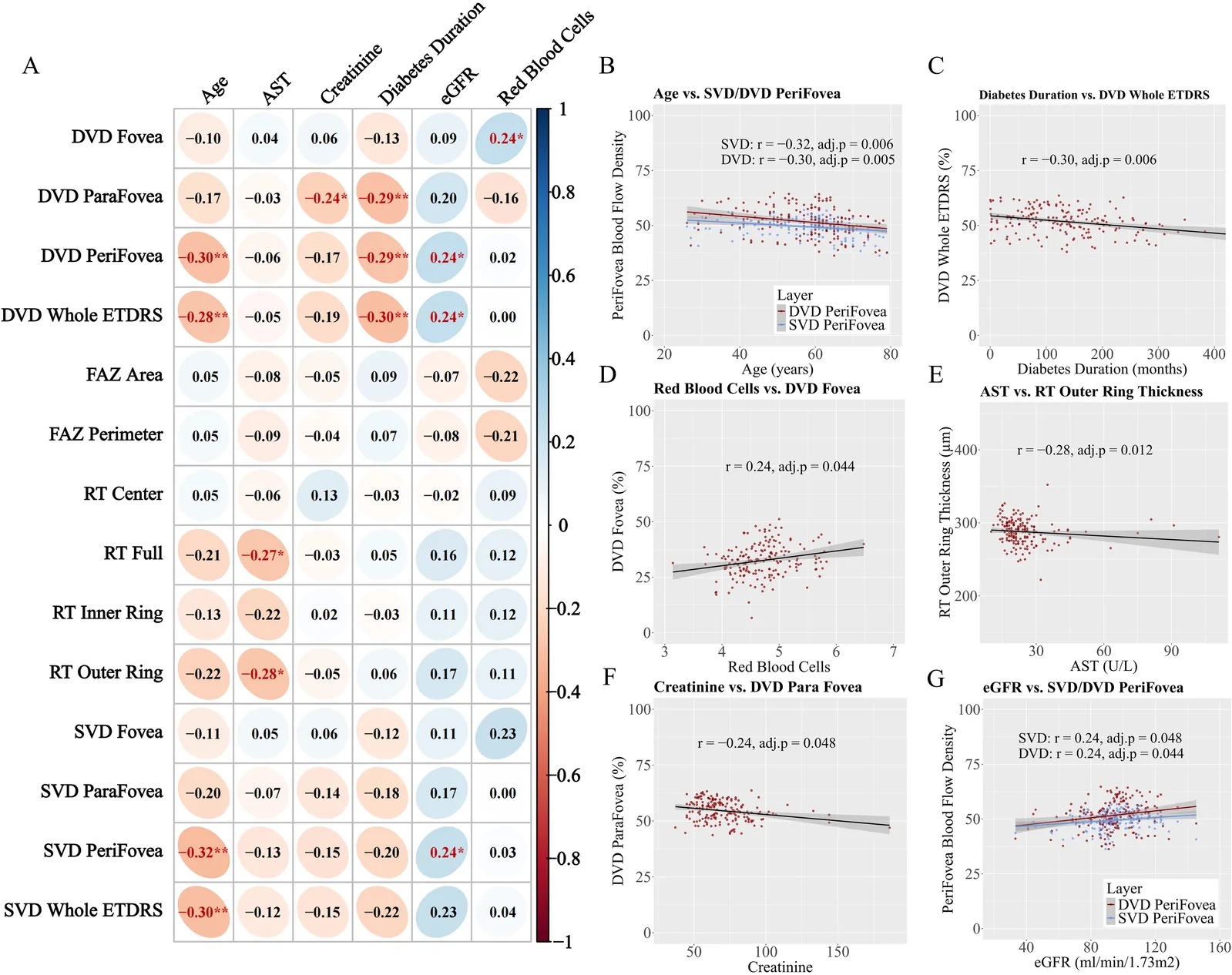
**(A)** Heatmap of spearman correlation coefficients between OCTA parameters and systemic factors that remained significant after with Benjamini-Hochberg correction. Blue indicates positive correlations, and red indicates negative correlations; B-G: Scatter plots showing representative significant correlations: **(B)** Age vs. superficial (SVD) and deep (DVD) vessel density in perifovea; **(C)** Diabetes duration vs. DVD in whole ETDRS; **(D)** RBC count vs. DVD in fovea. **(E)** AST vs. retinal thickness in the outer ring; **(F)** Serum creatinine vs. DVD in parafovea; **(G)** eGFR vs. SVD/DVD in perifovea. “*” indicates P < 0.05, “**”indicates P < 0.01.

In terms of hematological and renal biomarkers, DVD in fovea showed a positive correlation with red blood cell (RBC) count (r = 0.24, P = 0.044) (**Figure 2D**). DVD in parafovea was negatively associated with serum creatinine (r = -0.24, P = 0.048) (**Figure 2F**), and both SVD and DVD in perifovea were positively correlated with eGFR (r = 0.24 for both; p < 0.05) (**Figure 2G**). In addition, retinal thickness (RT) in the outer ring region was negatively correlated with aspartate aminotransferase (AST) levels (r = -0.28, P = 0.012) (**Figure 2E**). No significant correlations were observed between foveal avascular zone (FAZ) parameters and any systemic variables.

However, after adjustment for age, sex, and diabetes duration in multivariable linear regression models, these associations were no longer statistically significant.

### Feature selection

A total of 193 OCTA features and 31 systemic blood biomarkers were included for further data analysis (**Figure 3**). LASSO regression with cross-validation was applied to identify candidate markers associated with WKF. This procedure selected blood HbA1c and HDL-c and OCTA parameters such as Nasal inner superficial vessel density (NI-SVD, %) and inferior outer-ring retinal thickness (OI-RT, ILM-RPE, μm), and Central inner macular volume (C-IV, ILM-IPL, mm³). Bootstrap resampling demonstrated that NI-SVD had the highest selection frequency among the 193 OCTA features (66.3%), followed by C-IV (28.5%), whereas OI-RT ranked fifth (19.5%) (**Supplementary Figure S1**). Age, sex, and diabetes duration were selected as clinically relevant covariates and were included in all multivariable Cox proportional hazards models.

**Figure 3:**
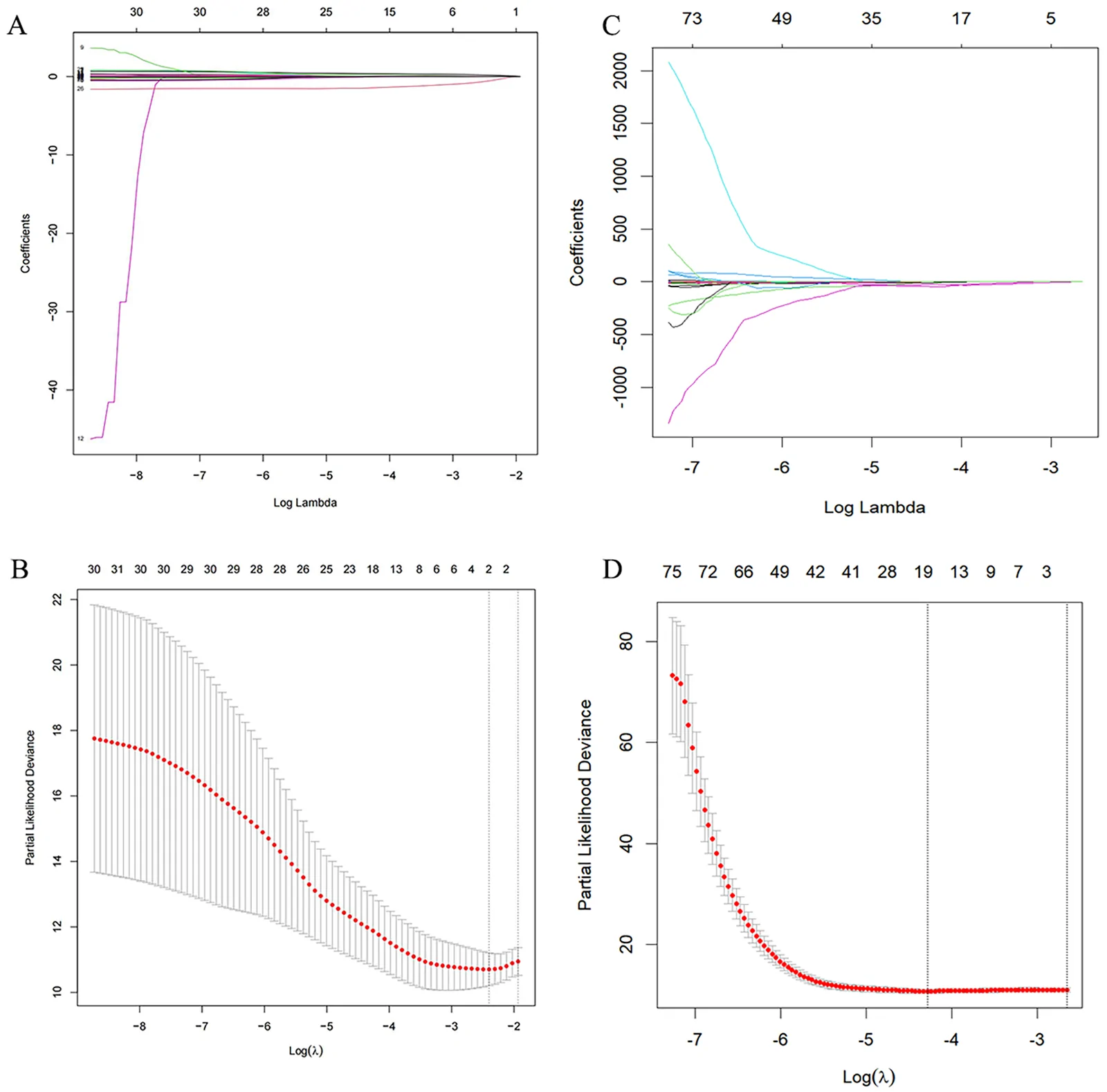
LASSO variable selection for blood biomarkers and OCTA parameters. **(A)** Coefficient profiles of blood biomarkers plotted against the log-transformed penalty parameter (log λ). **(B)** Ten-fold cross-validation curve for blood biomarkers. The red dots represent the mean cross-validated partial likelihood deviance, and the vertical bars indicate ±1 standard error. The two vertical dashed lines correspond to the minimum deviance (λ_min) and the most regularized model within 1 standard error of the minimum (λ_1se). **(C)** Coefficient profiles of OCTA parameters plotted against log λ. **(D)** Ten-fold cross-validation curve for OCTA parameters, with red dots indicating mean cross-validated partial likelihood deviance and error bars representing ±1 standard error. The optimal penalty parameter was selected according to λ_min.

### Multivariable cox analysis with and without early eGFR slope

In multivariable Cox proportional hazards models adjusting for clinical covariates and baseline eGFR (Model 2, n = 180), NI-SVD remained independently associated with WKF (HR 1.10, 95% CI 1.02-1.19, P = 0.013). C-IV was also significantly associated with WKF (HR 0.68 per SD, 95% CI 0.47-0.99, P = 0.042), whereas OI-RT showed no statistically significant association (HR 1.02, 95% CI 0.99-1.04, P = 0.165) (**Table 2**).

**Table 2:** Multivariable cox analysis with and without early eGFR slope.

Characteristic	Model 1 (n = 180)	P value	Model 2: (n = 180)	P value	Model 3 (n = 141)	P value
	HR (95% CI)		HR (95% CI)		HR (95% CI)	
Sex						
Female	ref.		ref.		ref.	
Male	1.38 (0.65-2.94)	0.402	1.43 (0.67-3.06)	0.354	2.01 (0.83-4.88)	0.124
Age, years	1.03 (0.99-1.07)	0.153	1.04 (0.99-1.09)	0.123	1.03 (0.98-1.08)	0.265
Diabetes duration, months	1.00 (1.00-1.01)	0.124	1.00 (1.00-1.01)	0.098	1.00 (1.00-1.01)	0.087
HbA1c, %	1.53 (1.24-1.90)	<0.001	1.53 (1.23-1.90)	<0.001	1.74 (1.33-2.29)	<0.001
HDL-c, mmol/L	0.06 (0.01-0.28)	<0.001	0.05 (0.01-0.26)	<0.001	0.37 (0.07-2.14)	0.269
NI-SVD, %	1.10 (1.02-1.19)	0.013	1.10 (1.02-1.19)	0.013	1.11 (1.02-1.21)	0.021
OI-RT, per μm	1.01 (0.99-1.04)	0.190	1.02 (0.99-1.04)	0.165	1.01 (0.99-1.04)	0.274
C-IV, per SD	0.68 (0.47-0.99)	0.045	0.68 (0.47-0.99)	0.042	0.67 (0.43-1.04)	0.077
Baseline eGFR, mL/min/1.73 m^2^	——	——	1.01 (0.99-1.03)	0.494	——	——
Early eGFR slope, per 1 mL/min/1.73 m^2^/year decrease	——	——	——	——	0.70 (0.54-0.89)	0.004

Model 1: Included age, sex, diabetes duration, HbA1c, HDL-c, and OCTA parameters. Model 2: Additionally adjusted for baseline eGFR. Model 3: A 12-month landmark model additionally included early eGFR slope (calculated using linear mixed-effects models) and was restricted to participants with available longitudinal eGFR measurements (n = 141). HR, hazard ratio; CI, confidence interval; eGFR, estimated glomerular filtration rate; HbA1c, glycated hemoglobin; HDL-c, high-density lipoprotein cholesterol; NI-SVD, nasal inner superficial vessel density; OI-RT, inferior outer-ring retinal thickness (ILM-RPE); C-IV, central inner macular volume (ILM-IPL, central 1-mm zone); NPDR, nonproliferative diabetic retinopathy; PDR, proliferative diabetic retinopathy. Hazard ratios for C-IV are expressed per 1-standard deviation (SD) increase (SD = 0.007mm3), whereas other continuous OCTA parameters are reported per original measurement unit.

Among the 180 patients, 141 participants who had at least two eGFR measurements and remained free of WKF within the first 12 months, with a total follow-up duration exceeding 12 months were included in the longitudinal eGFR trajectory and 12-month landmark analyses. Baseline characteristics of participants included and excluded from the slope analysis are shown in **Supplementary Table S1**.

Early eGFR slope (mean ± SD, -1.53 ± 1.38 mL/min/1.73 m^2^/year) during the first 12 months was estimated using a linear mixed-effects model. In the 12-month landmark analysis, early eGFR slope remained inversely associated with subsequent WKF (HR 0.70, 95% CI 0.54-0.89, p=0.004) (**Table 2**, model 3). NI-SVD remained significantly associated with subsequent WKF (HR 1.11, 95% CI 1.02-1.21, p=0.021), whereas C-IV was not statistically significant (HR 0.67, 95% CI 0.43-1.04, p=0.077) (**Table 2**).

### Sensitivity analysis

In sensitivity analyses with additional adjustment for baseline eGFR, ln(UACR), and MAP, the association between baseline NI-SVD and WKF remained consistent both in the full cohort (n = 180: HR 1.11, 95% CI 1.02 - 1.21, P = 0.012; **Supplementary Table S2** Sensitivity Model A) and in the slope-analysis sub-cohort using a 12-month landmark design (n = 141: HR, 1.09; 95% CI, 1.00-1.19; P = 0.042; **Supplementary Table S2** Sensitivity 12-Month Landmark Model B). Image quality metrics were well-balanced between WKF event and non-event groups (Overall 66.75 ± 7.67; WKF Events 66.47 ± 7.88 vs. Non-events 66.84 ± 7.63, P = 0.785) or QI (Overall 8.08 ± 0.96; WKF Events 8.12 ± 1.02 vs. Non-events 8.07 ± 0.95, P = 0.784; **Supplementary Table S3**). In sensitivity analyses, baseline NI-SVD maintained a consistent and statistically significant association with long-term WKF risk across all evaluated conditions (**Supplementary Table S4**), including after excluding DME cases (HR = 1.10, 95% CI: 1.01-1.20, P = 0.024), controlling for DR severity grade (HR = 1.11, 95% CI: 1.02-1.21, P = 0.012), and using the prespecified right eye (HR = 1.10, 95% CI: 1.01-1.19, P = 0.025), and further adjusting for AL (HR = 1.10, 95% CI: 1.01-1.20, P = 0.024). Multivariable restricted cubic spline analysis revealed a linear, monotonic positive association between baseline NI-SVD and WKF risk (P_overall = 0.016, P_non-linearity = 0.202; **Supplementary Figure S2**), with each 1-SD increase in NI-SVD associated with a 2.24-fold higher hazard of WKF (HR = 2.24, 95% CI: 1.29-3.88, P = 0.004).

### Exploratory mediation analysis

An exploratory mediation analysis was performed to assess whether early eGFR slope mediated the association between NI-SVD and WKF. Although NI-SVD showed a trend-level association with early eGFR slope and early eGFR slope was strongly associated with WKF, the indirect effect of NI-SVD on WKF through early eGFR slope did not reach statistical significance based on bootstrap confidence intervals (indirect HR = 1.02, 95% CI: 0.996-1.050).

## Discussion

In this longitudinal cohort of patients with type 2 diabetes, we found that baseline retinal OCTA parameters-particularly parafoveal superficial vessel density (NI-SVD)-were independently associated with subsequent worsening kidney function (WKF). This association persisted after adjustment for traditional metabolic and hemodynamic risk factors and remained statistically significant even after incorporating early eGFR trajectory into the model. In contrast, most cross-sectional associations between OCTA parameters and systemic biomarkers were attenuated after multivariable adjustment. These findings suggest that retinal microvascular alterations reflect a dimension of renal risk that is not adequately captured by conventional metabolic and renal biomarkers or short-term renal functional change.

Several cross-sectional associations were observed at baseline. Superficial and deep vessel density were inversely associated with age and diabetes duration, consistent with prior reports demonstrating progressive capillary rarefaction and perfusion decline with longer disease exposure (27–29). Associations were also observed between regional vessel density and renal biomarkers such as serum creatinine and eGFR, findings that align with earlier cross-sectional studies linking reduced retinal perfusion to lower eGFR in diabetic populations (30, 31). However, after adjustment for age, sex, and diabetes duration, these correlations no longer remained statistically significant. This attenuation indicates that shared upstream determinants-particularly cumulative glycemic exposure and aging-likely account for much of the observed covariance. Importantly, this pattern underscores a limitation of cross-sectional designs: retinal and renal parameters may appear linked at a single time point because both are downstream consequences of long-standing microvascular injury. The disappearance of statistical significance after adjustment therefore does not weaken the biological relevance of OCTA; rather, it clarifies that retinal microvascular metrics are not simple reflections of contemporaneous serum values.

The longitudinal findings are more informative. NI-SVD remained independently associated with WKF after comprehensive adjustment, including age, sex, glycemic control, lipid profile, diabetes duration and baseline eGFR. While prior OCTA studies in diabetes have largely focused on cross-sectional renal status or DR severity, prospective evidence remains scarce and is restricted to optic nerve head perfusion (32–35). Our findings extend this literature by demonstrating that macular superficial vessel density is associated with clinically relevant renal endpoints over time. The persistence of NI-SVD after adjustment suggests that parafoveal superficial microvasculature captures microvascular alterations relevant to renal risk that are not fully reflected by conventional risk factors. This association remained consistent across multiple sensitivity analyses, including accounting for image quality metrics, excluding DME cases, controlling for baseline DR severity grade or AL, and restricting to a prespecified single eye. The restricted cubic spline analysis further showed a linear, monotonic positive association between NI-SVD and WKF risk.

A notable finding was that higher NI-SVD was associated with increased renal risk. Although reduced vessel density is often interpreted as capillary loss, OCTA-derived density reflects flow signal rather than absolute vessel number (36). In diabetes, microvascular injury involves not only capillary dropout but also impaired autoregulation and altered perfusion dynamics. The superficial retinal capillary plexus supplies the inner retinal layers, which rely directly on the retinal microvasculature rather than choroidal diffusion and are therefore particularly sensitive to metabolic stress and hypoxia. In early microangiopathy, dysregulated flow in this metabolically active region may precede structural rarefaction (37). Specifically, this counterintuitive elevation in baseline flow signal may reflect early retinal microvascular hyperperfusion, a frequently reported compensatory state in early diabetic microvasculopathy, or the presence of intraretinal microvascular abnormalities (IRMAs) and arteriovenous shunt vessels formed during early vascular remodeling (38–40). Compensatory shunt vessels and their derived IRMAs are predominantly localized within the SVC (41–43). Such structural and hemodynamic dysregulations produce a transiently elevated or erratic OCTA flow signal prior to overt capillary loss. Therefore, elevated NI-SVD may thus represent abnormal hyperperfusion, compensatory remodeling or early microvascular instability within the superficial plexus rather than preserved vascular integrity. The persistence of this association after adjustment for conventional risk factors and early eGFR slope suggests that NI-SVD may reflect a microvascular instability profile associated with renal vulnerability. However, these mechanisms remain hypothesis-generating, because OCTA measurements cannot distinguish increased perfusion from altered flow dynamics, vascular recruitment, or other sources of increased flow signal.

The analysis of early eGFR slope was restricted to 141 participants with sufficient follow-up (≥12 months and ≥2 eGFR measurements), leaving 39 participants excluded from slope modeling. Although some metabolic variables differed between groups, these were adjusted for in multivariable models and did not materially alter the association between NI-SVD and WKF. Early eGFR slope was, as expected, strongly associated with renal outcomes, consistent with evidence that renal function trajectory provides prognostic information beyond single baseline measurements (44, 45). Incorporating early eGFR slope into the multivariable model attenuated but did not eliminate the association between NI-SVD and WKF. This finding is clinically meaningful. Early slope quantifies short-term functional decline, whereas NI-SVD reflects baseline microvascular structure and perfusion. The persistence of NI-SVD after adjustment for slope indicates that retinal microvascular abnormalities are not merely downstream manifestations of early measurable renal decline. Rather, structural microvascular vulnerability and functional trajectory appear to represent overlapping but partially independent dimensions of renal risk. The exploratory mediation analysis further supports this interpretation, as no statistically significant indirect pathway through early eGFR slope was identified. While limited statistical power cannot be excluded, these findings argue against a single linear pathway linking retinal microvascular change to renal outcome solely through early functional deterioration.

The regional specificity of the association also warrants consideration. Given the heterogeneous distribution of retinal nerve cells and uneven oxygen consumption across retinal layers and regions, macular blood flow exhibits pronounced spatiotemporal heterogeneity (15, 46). The stronger association of NI-SVD compared with other OCTA parameters in our cohort supports the importance of spatially resolved microvascular assessment. From a translational perspective, these layer-segmented, region-specific findings may offer mechanistically interpretable correlates that complement hypothesis-free retinal AI models for DKD risk screening.

Several limitations should be acknowledged. The modest sample size, particularly for trajectory and mediation analyses, may have limited statistical power. The single-center design may restrict generalizability. Given these constraints, this study was designed to evaluate risk associations rather than to construct a formal clinical prediction model; accordingly, internal validation, calibration curves, and decision-curve analyses were not performed to avoid model overfitting. Retinal imaging was obtained only at baseline, precluding evaluation of longitudinal retinal change. Early eGFR slope was model-derived rather than directly observed. In addition, the mechanistic interpretation of higher NI-SVD remains speculative because OCTA measures flow-related signal rather than direct capillary perfusion or vascular function. As an observational study, causal inference cannot be established. Replication in larger multicenter cohorts with repeated retinal imaging and longer follow-up will be essential. Future studies combining longitudinal OCTA, multimodal retinal imaging, and mechanistic biomarkers may help determine whether the NI-SVD phenotype represents an early, reversible perfusion abnormality or a marker of subsequent microvascular rarefaction.

## Conclusions

In summary, baseline parafoveal superficial vessel density was independently associated with worsening kidney function in patients with type 2 diabetes. This association was not explained by early renal functional decline, suggesting that retinal microvascular alterations capture microvascular alterations relevant to renal risk beyond conventional biomarkers and short-term eGFR trajectory. These findings support further investigation of retinal OCTA as a potential noninvasive marker of renal risk.

## Data Availability

The datasets presented in this article are not readily available because the datasets generated and/or analyzed during the current study are not publicly available due to the privacy concerns of the participants but are available from the corresponding author upon reasonable request. Requests to access the datasets should be directed to Yifei Zhang, feifei-a@163.com.

## Supplementary material

Supplementary Figure 1 Feature selection frequency spectrum across 1,000 bootstrap iterations.Ranking of the top 10 candidate features according to their selection frequencies evaluated by executing the full 10-fold cross-validated regularized Cox regression pipeline across 1,000 bootstrap samples. Bootstrap resampling demonstrated that NI-SVD had the highest selection frequency among the 193 OCTA features (66.3%), followed by C-IV (28.5%), whereas OI-RT ranked fifth (19.5%). The red dashed vertical line represents the 50% selection frequency threshold commonly used to identify stable predictors. Abbreviations: NI-SVD, nasal inner parafoveal superficial vessel density; C-IV, central inner macular volume (ILM-IPL, central 1-mm-diameter zone); IR-SI-Diff-GCC, inner-retina superior-minus-inferior intra-eye thickness difference (ILM-IPL) from GCC scan; TI-DVD, temporal inner-ring deep vessel density; OI-RT, inferior outer-ring retinal thickness; IR-Sup-RT-GCC, inner-retina superior average retinal thickness (ILM-IPL) from GCC scan; C-IR-RT, central inner-retina retinal thickness (ILM-IPL, central 1-mm-diameter zone); F-DVD, foveal deep vessel density; F-SVD, foveal superficial vessel density; OR-SI-Diff-GCC, outer-retina superior-minus-inferior intra-eye thickness difference (IPL-RPE) from GCC scan.

Supplementary Figure 2 Restricted cubic spline (RCS) plot of baseline NI-SVD and the hazard of worsening kidney function (WKF).The solid blue line represents the continuous multivariable-adjusted hazard ratios (HRs), and the light blue shaded area depicts the 95% confidence intervals. The horizontal dashed line indicates the reference hazard ratio (HR = 1.0, centered at the median baseline NI-SVD). The model was dynamically adjusted for diabetes duration, mean arterial pressure, baseline eGFR, log-transformed UACR, OCTA Signal Strength Index (OCTASSI), Overall Quality Index (OverallQI), and baseline diabetic retinopathy severity class. P for overall association = 0.016, P for non-linearity = 0.202.
