# Supplementary material for: Retinal OCTA parameters and the risk of worsening kidney function in patients with type 2 diabetes: a longitudinal cohort study
Source: Front Endocrinol (Lausanne). 2026 Sep 14;17:1924491. doi: 10.3389/fendo.2026.1924491 (PMC13616706; doi:10.3389/fendo.2026.1924491)
Supplement: Supplementary file 1 [file Table1.docx]

| Supplementary Table 1. Baseline characteristics of patients included vs excluded from the early eGFR slope analysis | | | | |
| --- | --- | --- | --- | --- |
| Characteristic | level | Excluded from slope analysis | Included in slope analysis | p |
|  |  | n=39 | n=141 |  |
| Outcome (%) | no event | 31 (79.49) | 107 (75.89) | 0.797 |
|  | eGFR decline≥20% | 8 (20.51) | 34 (24.11) |  |
| Sex (%) | Female | 19 (48.72) | 63 (44.68) | 0.790 |
|  | Male | 20 (51.28) | 78 (55.32) |  |
| Age, years (median [IQR]) |  | 56.00 [49.00, 66.00] | 60.00 [51.00, 65.00] | 0.954 |
| Diabetes Duration, months (median [IQR]) |  | 125.30 [65.95, 180.60] | 128.20 [68.73, 176.70] | 0.780 |
| MAP, mmHg (median [IQR]) |  | 91.33 [84.50, 99.67] | 89.33 [83.00, 97.33] | 0.356 |
| HbAlc (median [IQR]) |  | 7.60 [6.70, 8.95] | 7.20 [6.60, 8.10] | 0.150 |
| HDL-c (median [IQR]) |  | 1.09 [0.78, 1.40] | 1.18 [1.03, 1.41] | 0.030 |
| NI-SVD, % (median [IQR]) |  | 49.86 [46.66, 52.03] | 51.70 [48.81, 54.48] | 0.007 |
| OI-RT, μm (median [IQR]) |  | 269.30 [257.45, 278.75] | 272.70 [261.80, 282.10] | 0.144 |
| C-IV, mm^3^ (median [IQR]) |  | 0.04 [0.04, 0.04] | 0.04 [0.04, 0.04] | 0.164 |
| eGFR, mL/min/1.73 m² (median [IQR]) |  | 94.12 [88.50, 105.31] | 94.27 [85.53, 103.68] | 0.339 |
| UACR, mg/mmol (median [IQR]) |  | 2.50 [0.99, 5.96] | 2.50 [1.19, 4.72] | 0.836 |
| Staging of Retinopathy (%) | Normal | 15 (38.46) | 56 (39.72) | 0.122 |
|  | Mild NPDR | 11 (28.21) | 47 (33.33) |  |
|  | Moderate NPDR | 12 (30.77) | 30 (21.28) |  |
|  | Severe NPDR | 0 (0.00) | 8 (5.67) |  |
|  | PDR | 1 (2.56) | 0 (0.00) |  |
| Abbreviations: WKF, worsening kidney function; MAP, mean arterial pressure; UACR, urinary albumin-to-creatinine ratio; eGFR, estimated glomerular filtration rate; HbA1c, glycated hemoglobin; HDL-c, high-density lipoprotein cholesterol; NI-SVD, nasal inner superficial vessel density; C-IV, central inner macular volume (ILM–IPL, central 1-mm zone); OI-RT, inferior outer-ring retinal thickness (ILM–RPE); NPDR, nonproliferative diabetic retinopathy; PDR, proliferative diabetic retinopathy. | | | | |
|  |  |  |  |  |

| Supplementary Table S2. Sensitivity analysis of the association between NI-SVD and worsening kidney function after additional adjustment for baseline eGFR, ln(UACR), and MAP | | | | |
| --- | --- | --- | --- | --- |
| Characteristic | Sensitivity Model A (n = 180) | P value | Sensitivity 12-Month Landmark Model B (n=141) | P value |
|  | HR (95% CI) |  | HR (95% CI) |  |
| Diabetes duration, months | 1.00(1.00-1.01) | 0.023 | 1.01(1.00-1.01) | 0.007 |
| MAP, mmHg | 1.02(0.99-1.05) | 0.204 | 1.03(0.97-1.07) | 0.203 |
| HbA1c, % | 1.52(1.24-1.86) | <0.001 | 1.68(1.29-2.19) | <0.001 |
| HDL-c, mmol/L | 0.13(0.04-0.47) | 0.002 | 0.31(0.06-1.55) | 0.155 |
| NI-SVD, % | 1.11(1.02-1.21) | 0.012 | 1.09(1.00-1.19) | 0.042 |
| Baseline eGFR, mL/min/1.73 m^2^ | 1.00(0.98-1.02) | 0.815 | 1.02(1.00-1.04) | 0.102 |
| ln(UACR[mg/mmol]) | 1.17(0.93-1.48) | 0.180 | 1.04(0.79-1.37) | 0.764 |
| Early eGFR slope, per 1 mL/min/1.73 m^2^/year increase | - | - | 0.56(0.41-0.77) | <0.001 |
| Abbreviations: HR, hazard ratio; CI, confidence interval; MAP, mean arterial pressure; NI-SVD, nasal inner-circle superficial vessel density; eGFR, estimated glomerular filtration rate; UACR, urine albumin-to-creatinine ratio. UACR was natural log-transformed before inclusion in the Cox regression model. | | | | |

| Supplementary Table S3. Quality Index Distributions | | | | |
| --- | --- | --- | --- | --- |
| Metric | Total Cohort (N=180) | WKF Events (N=42) | Non-Events (N=138) | *P* Value |
| Signal Strength Index (SSI) | 66.75±7.67 | 66.47 ±7.88 | 66.84 ±7.63 | 0.785 |
| Quality Index (QI) | 8.08±0.96 | 8.12 ±1.02 | 8.07 ± 0.95 | 0.784 |

| Supplementary Table S4. Sensitivity Cox Models for NI-SVD | | | |
| --- | --- | --- | --- |
| Model Description | Evaluated Cohort / Condition | HR (95% CI) | P Value |
| Sensitivity Model 1 | Excluding DME cases ( N=177 ) | 1.10（1.01-1.20） | 0.024 |
| Sensitivity Model 2 | Adjusted for DR severity Grade | 1.11（1.02-1.21） | 0.012 |
| Sensitivity Model 3 | Prespecified Single Eye (Right Eye) | 1.10（1.01-1.19） | 0.025 |
| Sensitivity Model 4 | Adjusted for axial length | 1.10（1.01-1.20） | 0.024 |
| Note: Models were adjusted for baseline eGFR, ln(UACR) , Mean Arterial Pressure (MAP), HbA1c, HDL-c, and Diabetes Duration. HR = Hazard Ratio; CI = Confidence Interval. | | | |
